# Effectiveness and Safety of Herbal Medicine for Atopic Dermatitis: An Overview of Systematic Reviews

**DOI:** 10.1155/2020/4140692

**Published:** 2020-07-17

**Authors:** Chan-Young Kwon, Boram Lee, Suran Kim, Jaesuk Lee, Minjung Park, Namkwen Kim

**Affiliations:** ^1^Department of Oriental Neuropsychiatry, Dong-eui University College of Korean Medicine, 62 Yangjeong-ro, Busanjin-gu, Busan 47227, Republic of Korea; ^2^Clinical Medicine Division, Korea Institute of Oriental Medicine, 1672 Yuseongdae-ro, Yuseong-gu, Daejeon 34054, Republic of Korea; ^3^Guideline Center for Korean Medicine, National Institute for Korean Medicine Development, 173 Toegye-ro, Jung-gu, Seoul 04554, Republic of Korea; ^4^Department of Ophthalmology and Otolaryngology and Dermatology, School of Korean Medicine, Pusan National University, 20 Geumo-ro, Mulgeum-eup, Yangsan-si, Gyeongsangnam-do 50612, Republic of Korea

## Abstract

**Objectives:**

Herbal medicine (HM) is attracting attention for treating atopic dermatitis (AD). This overview was conducted to summarize and critically evaluate the current systematic reviews (SRs) on HM for the treatment of AD.

**Methods:**

Through comprehensive searches, all relevant SRs on HM for AD published until May 2020 were included. The quality of included SRs was assessed using the AMSTAR-2 tool. Moreover, original randomized controlled trials (RCTs) included in the SRs were resynthesized to investigate the efficacy and safety of oral HM for AD. The quality of evidence for the main findings was evaluated using the GRADE approach.

**Results:**

Nine SRs were included in this overview. HM showed significantly better efficacy in terms of total effective rate (TER), itching and sleep symptom scores, quality of life, and the dose of topical treatment used compared with placebo. HM as a monotherapy and/or an adjunctive therapy to conventional medication (CM) showed significantly better results on the efficacy, symptom relief, and some laboratory parameters related to the inflammatory response. The methodological quality was generally low. When 58 original RCTs were reanalyzed, HM showed significantly lower SCORing Atopic Dermatitis (SCORAD) score and higher TER than the placebo or CM. In terms of the safety profile, HM was not significantly different from the placebo and was better than CM. The quality of evidence ranged from “moderate” to “very low.”

**Conclusion:**

The results suggested that HM as a monotherapy or an adjunctive therapy is promising for the treatment of AD. However, due to low methodological quality and low quality of evidence, further rigorous, well-designed, high-quality SRs, and RCTs are needed to make clinical recommendations on HM use.

## 1. Introduction

Atopic dermatitis (AD) is a common, chronic, inflammatory, and refractory skin disease characterized by itching, dry skin, skin redness, and thickened skin. The prevalence of this disease is known to be around 2.1%–4.9% worldwide [1]. In addition to genetic causes, environmental factors such as the history of prototypical infections (e.g., hepatitis and tuberculosis), relations with microflora, duration of breastfeeding, and social position of the parents are considered to be relevant [2]. Today, topical glucocorticosteroids, calcineurin inhibitors, tacrolimus, and pimecrolimus as well as some adjuvant therapies including ultraviolet (UV) irradiation, lifestyle modification, antimicrobial therapy, systemic anti-inflammatory treatment, and immunotherapy are recommended for the treatment of AD based on the international clinical guidelines [3–5]. However, the efficacy of a highly standardized treatment strategy is insufficient, and highly individualized treatment is recommended for the management of AD, emphasizing the need for a tailored multimodal strategy [4]. In this regard, attempts to use complementary and integrative medicine (CIM) in the management of AD are increasing [6].

East Asian traditional medicines (EATMs) such as traditional Chinese medicine, traditional Korean medicine, and Kampo medicine have been used for health care in Asia for thousands of years. Recently, valuable CIM approaches such as herbal medicine (HM) are known to be popular not only in Asian countries but also in Western countries. Although solid evidence is still needed, some HMs of both topical use and oral administration have been shown to be effective in skin diseases including AD [7–9]. Based on these accumulating evidences, in the Republic of Korea, a clinical practice guideline (CPG) on HM treatment for AD was developed and published in 2016 [10].

In the treatment of diseases with complex pathological mechanisms such as AD, HM with the so-called “multicomponent, multitarget, and multipathway” characteristic can be a suitable candidate [11]. Clinical evidences supporting the efficacy and safety of HM as treatment for AD have been extensively accumulated, and some systematic reviews (SRs) have summarized these clinical evidences. However, the evidence and methodological quality of SRs regarding the use of HM for AD has not yet been summarized and evaluated, and this should be conducted for clinical recommendations. Today, an overview of systematic reviews, or umbrella reviews, has been considered as a new research strategy that described the current body of SR evidence [12–14]. It provides a synthesis and integrates information from multiple studies to reduce the uncertainties in decision-making and to provide updated evidence, in situations where there are a large number and various qualities of available information [15].

This overview aimed to assess the methodological quality of the current SRs on the efficacy and safety of HM as a treatment for AD. Moreover, we aimed to reanalyze and synthesize original randomized controlled clinical trials (RCTs) from the included SRs on oral HM for AD to clarify its efficacy and safety and to evaluate the quality of evidence for the main findings.

## 2. Methods

We conducted the overview according to the guidelines stated in the Cochrane handbook [16]. Ethics approval was not required as this is an overview. The protocol of this overview was registered in PROSPERO (registration ID: CRD42020150475).

### 2.1. Search Strategy

One reviewer (CYK) conducted a comprehensive search on three English databases (Medline (via PubMed), EMBASE (via Elsevier), and the Cochrane Library), one Chinese database (the China National Knowledge Infrastructure), and one Korean database (the Oriental Medicine Advanced Searching Integrated System). The initial search date was June 14, 2019, and we conducted an updated search on May 24, 2020, to provide a more up-to-date and comprehensive evidence. There was no restriction on language, publication status (including gray literature), or publication country. In addition, the reference lists of the included reviews were hand-searched to identify additional relevant reviews. The following terms were used when searching PubMed: (Dermatitis, Atopic[MeSH] OR atopic[tiab]) AND (Drugs, Chinese Herbal[MeSH] OR Medicine, East Asian Traditional[MeSH] OR Herbal Medicine[MeSH] OR herbal[tiab]). The search strategy for each database is presented in Supplement 1.

### 2.2. Inclusion and Exclusion Criteria

This overview included SRs and/or meta-analyses that examined the efficacy and safety of HM on AD by analyzing RCTs, quasi-RCTs, and/or controlled clinical trials (CCTs) regardless of age, gender, race/ethnicity, and disease duration of AD patients. HM was compared with no treatment, usual care, or active controls such as conventional medication (CM) in the treatment of AD, or the benefit of HM combined with other treatments was compared with those of other treatments alone. In this study, HM included not only individual herbs but also prescriptions composed of various herbs, and studies on individual compounds extracted from herbs were also permitted. The following cases were excluded: (1) non-SRs, (2) SRs that provided a comparison between multiple HMs, and (3) SRs that analyzed the efficacy and safety of HM for nonhuman studies, for example, SR for animal studies.

### 2.3. Study Selection and Data Extraction

Two reviewers (CYK and BL) independently assessed whether the searched articles met the inclusion criteria. The screening of the titles and abstracts of the searched literature was conducted for first inclusion, and the full texts of all eligible studies were reviewed for final inclusion. In case of disagreement, they tried to resolve it through discussions.

Using a standardized data extraction form in Excel 2016, the following data were extracted from included SRs: first author's name, publication year, country, number of primary studies included, total sample size, search period, interventions of treatment group and control group, major outcomes and results from meta-analysis, safety data, details on the assessment of methodological quality, the author's conclusions, and cautions in the interpretation of results. Two reviewers (CYK and BL) independently extracted the abovementioned information from the included SRs, and in case of disagreement, they tried to resolve it through discussions.

### 2.4. Quality Assessment

The Assessing the Methodological Quality of Systematic Reviews- (AMSTAR-) 2 tool was used to assess the methodological quality of the included SRs by two independent reviewers (CYK and BL) [17]. This 16-item validated evaluation tool is used for evaluating the methodological quality of an SR. All 16 items were evaluated and rated as “yes,” “partially yes,” or “no” [17]. AMSTAR-2 does not generate an overall score; however, the overall quality of each SR was assessed and classified as either “high,” “moderate,” “low,” or “critically low” by referencing the critical weakness and flaw of each SR [17]. In case of disagreement in the result of quality assessment, the two reviewers (CYK and BL) tried to resolve it through discussions.

### 2.5. Data Analysis

#### 2.5.1. Qualitative Synthesis of Included SRs

In the first step, a qualitative synthesis of the included SRs was performed using the abovementioned extracted data. The data obtained from each SR was presented as odds ratio (OR) or risk ratio (RR) for dichotomous data, and mean difference (MD) or standardized mean difference (SMD) for continuous data, with 95% confidence intervals (CIs).

#### 2.5.2. Quantitative Synthesis of Original RCTs

In the second step, we obtained the full text of the original RCTs in the SRs included in this overview to comprehensively reevaluate the efficacy and safety of HM for AD using a meta-analysis, particularly the oral HM. In addition, data on the components of oral HM used were extracted from each RCT by the two independent reviewers (CYK and BL). For the outcomes including the scoring atopic dermatitis (SCORAD) score, total effective rate (TER), and the incidence of adverse events (AEs), a meta-analysis of the following comparisons was conducted: (1) oral HM versus placebo, (2) oral HM versus conventional medicine, and (3) oral HM combined with conventional medicine versus conventional medicine alone. The heterogeneity of effect measures between the studies was assessed using both the chi-square test and the *I*-squared statistic (*I*^*2*^). When the value of *I*^2^ is greater than 50% or 75%, the heterogeneity was considered to be substantial or high, respectively. When the heterogeneity was not high (*I*^2^ ≤ 75%) and when the number of studies included in each meta-analysis was less than five [18, 19], a fixed-effects model was used. Otherwise, a random-effects model was used. We used the Review Manager version 5.3 software (Cochrane, London, UK) to perform all statistical analyses.

### 2.6. Quality of Evidence

Using the grading of recommendations, assessment, development, and evaluation (GRADE) approach, two reviewers (CYK and BL) independently assessed the quality of evidence for the main findings from the quantitative synthesis of original RCTs. The GRADE method is a validated tool for evaluating the quality of evidence by assessing the following five key items: (1) risk of bias (RoB), (2) inconsistency, (3) indirectness, (4) imprecision of results, and (5) probability of publication bias. For the assessment of the RoB item, we reassessed the quality of primary RCTs within the SRs using Cochrane's RoB tool [20]. The quality of evidence for the main findings was judged as “very low,” “low,” “moderate,” or “high.” In case of disagreement, the two reviewers tried to resolve it through discussions.

## 3. Results

### 3.1. Description of Included Studies

**Figure 1:**
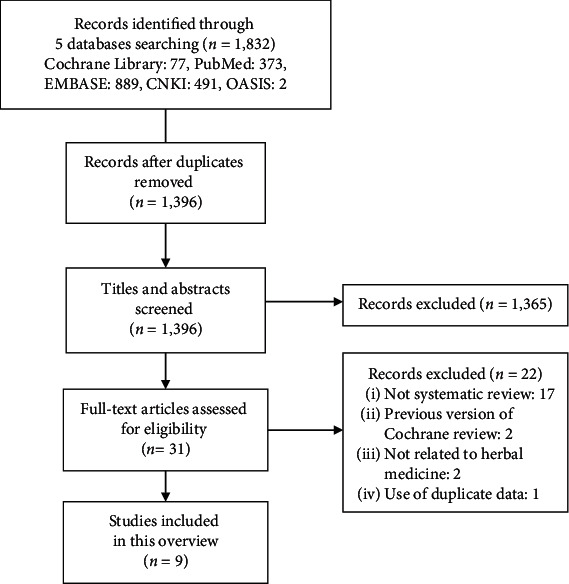
PRISMA flow chart of the study selection process.

We identified 1,832 studies by searching five databases. After removing duplications, the titles and abstracts of the remaining 1,396 studies were screened. Then, the full texts of 31 potentially relevant articles were reviewed for eligibility. Seventeen articles that were identified as non-SRs, two that were the previous version of Cochrane review, two that were not related to HM, and one that used the same data (dissertation) were excluded. Finally, a total of 9 SRs [7, 21–28] were included in this overview (Figure 1).

### 3.2. Study Characteristics

All nine SRs [7, 21–28] included in this overview conducted a meta-analysis of 6–37 RCTs. Four reviews [7, 22, 25, 27] were published in English, and the remaining five [21, 23, 24, 26, 28] were published in Chinese. There was a Cochrane review [22] comparing the efficacy and safety of oral and/or external HM with placebo, no treatment, or active controls. Among the remaining SRs, one [21] compared oral HM with oral antihistamines; one [7] compared oral HM with placebo, no treatment, or active controls; one [23] compared oral HM alone or in combination with CM with CM alone; one [24] compared oral or external HM in combination with CM with CM alone; one [25] compared EATM treatments including oral HM, acupuncture, and moxibustion with placebo or CM; one [26] compared oral Jinpi (meaning tonifying spleen) HM alone or in combination with CM with CM alone; one [27] compared Tripterygium agents alone or in combination with CM with CM alone; one [28] compared oral or external HM with CM or placebo. Among the outcomes meta-analyzed, TER was the most frequently used outcome in eight reviews [21–28], followed by recurrence rate in six reviews [21, 23, 25–28], and SCORAD score and serum level of immune-related substances such as immunoglobulin E (IgE), interleukin (IL)-*γ*, IL-4, and eosinophils (EOS) in four reviews [23, 26–28], respectively. The main characteristics of the included SRs are shown in Table 1.

### 3.3. Methodological Quality

According to the AMSTAR-2 checklist, all reviews specified the research question and inclusion criteria including the components of PICO and explained the selection of the study designs for inclusion. However, only two reviews [22, 27] preregistered the study protocol. The authors of most SRs [21–23, 25, 27, 28] independently performed the study selection and data extraction. Except for the Cochrane review [22], all other studies did not describe the excluded studies with reasons. Except for one study [21], which did not even describe the list of included RCTs, all other studies described the details of included RCTs adequately [7, 22, 25, 27] or insufficiently [23, 24, 26, 28]. Three reviews [21, 23, 24] that assessed the methodological quality of included RCTs using only the Jadad scale were evaluated using an unsatisfactory quality assessment tool. All other reviews using Cochrane's RoB tool were evaluated using a satisfactory quality assessment tool. With regard to the sources of funding for the included RCTs, only the Cochrane review [22] reported the details. Most reviews [7, 21, 22, 24, 25, 27, 28] included were considered to use the appropriate method for the statistical combination of results. The two reviews [23, 26] rated “no” did not provide a definition of the use of the random-effects model or the fixed-effects model or distinguish their use according to statistical heterogeneity. Three reviews [21, 22, 25] considered the effect of RoB of the included RCTs on the synthesis results. Among them, two reviews [21, 22] performed a sensitivity analysis according to methodological quality, and the other one [25] used a methodological quality as selection criteria for their analysis. Except for one review [21], all the remaining reviews noted the effect of RoB of the included RCTs on the reliability of their results. Except for the two reviews [23, 26], the remaining reviews did not show statistically significant heterogeneity in their meta-analysis or described an explanation to clarify the heterogeneity that occurred. With regard to reporting bias, except for one review [25], the investigation of publication bias was conducted. All included reviews had no potential sources of conflicts of interest. The overall quality of one Cochrane review [22] was high without a critical flaw. However, the remaining reviews had low or critically low quality (Table 2).

### 3.4. What Do the SRs Say about HM for AD?

#### 3.4.1. HM versus Placebo

Two reviews [7, 22] reported the efficacy of HM on AD compared with the placebo. Gu et al. [22] reported that oral or external HM showed better results in terms of TER (RR: 2.09, 95% CI: 1.32 to 3.32, 2 studies, 85 participants), itching score (which was rated using a visual analog scale (VAS)) (SMD: −1.53, 95% CI: −2.64 to −0.41, 2 studies, 94 participants), overall severity score (SMD: −0.88, 95% CI −1.67 to −0.09, 4 studies, 239 participants), and children's dermatology life quality index (CDLQI) score (MD: −2.50, 95% CI: −4.77 to −0.23, 1 study, 85 participants) compared with placebo. They planned to perform sensitivity analysis by excluding studies with low methodological quality; however, they failed to do due to the lack of enough studies. Tan et al. [7] reported that oral HM showed significant benefits compared with placebo based on the erythema score (SMD: −0.76, 95% CI: −1.05 to −0.47, 3 studies, 245 participants), surface damage score (SMD: −1.08, 95% CI: −1.59 to −0.56, 3 studies, 245 participants), itching score (MD: −1.10, 95% CI: −1.59 to −0.61, 1 study, 71 participants), sleep score (MD −0.80, 95% CI: −1.12 to −0.48, 1 study, 71 participants), CDLQI score (MD: −2.50, 95% CI: −4.77 to −0.23, 1 study, 85 participants), and a dose of topical treatment used (MD: −24.50, 95% CI: −27.92 to −21.08, 1 study, 91 participants). However, both of these reviews were based on a small number of and low-quality RCTs; therefore, caution should be taken when interpreting our results (Table 1).

#### 3.4.2. HM versus CM

*(1) Efficacy and Symptom Relief*. In most reviews [7, 21–24, 26–28], HM as a monotherapy and/or an adjunctive therapy to CM showed significantly better results on the efficacy and symptom relief. Gong [21] reported that oral HM showed significantly better results on TER compared with oral antihistamines (OR: 5.64, 95% CI: 4.07 to 7.81, 11 studies, 1479 participants). Through sensitivity analysis according to study quality, and sensitivity analysis between the random-effects model and fixed-effect model, consistent conclusions were reached showing that the conclusions of the study were credible. Gu et al. [22] reported that oral or external HM showed significantly better results on TER (RR: 1.43, 95% CI: 1.27 to 1.61, 21 studies, 1868 participants), itching score (SMD: −0.83, 95% CI: −1.43 to −0.22, 7 studies, 465 participants), and overall severity (SMD: −0.97, 95% CI: −1.23 to −0.71, 15 studies, 1062 participants) compared with CM. They planned to perform sensitivity analysis by excluding studies with low methodological quality; however, they failed to do due to the lack of data. Tan et al. [7] reported that oral HM as an adjunctive therapy to CM showed significantly better overall severity score (MD: −2.56, 95% CI: −3.46 to −1.66, 1 study, 98 participants) compared with CM alone. Yang et al. [23] reported that oral HM as a monotherapy or an adjunctive therapy to CM showed significantly better results on TER (OR: 4.86, 95% CI: 3.44 to 6.87, 13 studies, 1232 participants) and SCORAD score (MD: −15.51, 95% CI: −20.15 to −10.87, 4 studies, 390 participants) compared with CM alone. Ma et al. [24] reported that oral or external HM as an adjunctive therapy to CM showed significantly better results on cure rate (OR: 2.94, 95% CI: 2.08 to 4.16, 11 studies, 757 participants) and TER (OR: 4.86, 95% CI: 3.13 to 7.56, 12 studies, 733 participants). Liu et al. [26] reported that oral Jinpi HM as a monotherapy or an adjunctive therapy to CM showed significantly better results on TER (OR: 4.05, 95% CI: 3.27 to 5.03, 30 studies, 2333 participants), SCORAD score (MD: −9.82, 95% CI: −13.31 to −6.33, 15 studies, 1282 participants), Eczema Area and Severity Index (EASI) score (MD: −2.80, 95% CI: −3.54 to −2.07, 3 studies, 232 participants), and itching score (MD: −0.79, 95% CI: −1.10 to −0.47, 7 studies, 448 participants) compared with CM alone. Moreover, on TER, oral Jinpi HM as a monotherapy (OR: 4.81, 95% CI: 3.63 to 6.36, 21 studies, 1355 participants) and an adjunctive therapy to CM (OR: 2.94, 95% CI: 2.11 to 4.11, 9 studies, 832 participants) showed significantly better results compared with CM alone. Liu et al. [27] reported that Tripterygium agents as a monotherapy or an adjunctive therapy to CM showed significantly better results on TER (RR: 1.59, 95% CI: 1.26 to 2.00, 13 studies, 1361 participants) compared with CM alone. However, Tripterygium agents as a monotherapy did not show significant benefits compared with CM (RR: 1.19, 95% CI: 0.96 to 1.48, 4 studies, 367 participants), while Tripterygium agents as an adjunctive therapy to CM showed significant benefits (RR: 1.78, 95% CI: 1.40 to 2.25, 9 studies, 994 participants) on TER. Wang et al. [28] showed that oral or external HM showed significantly better results on cure rate (RR: 1.79, 95% CI: 1.35 to 2.39, 16 studies, 1727 participants), TER (RR: 1.19, 95% CI: 1.08 to 1.31, 16 studies, 1727 participants), and SCORAD score (MD: −14.67, 95% CI: −19.52 to −9.82, 4 studies, 292 participants) compared with CM (Table 1).

*(2) Recurrence Rate*. For the recurrence rate, mixed results were reported according to reviews. Gong [21] reported that oral HM showed a significantly lower recurrence rate than oral antihistamines (OR: 0.38, 95% CI: 0.29 to 0.49, 11 studies, 1436 participants). Yang et al. [23] reported that oral HM as a monotherapy or an adjunctive therapy to CM showed significantly lower recurrence rate (OR: 0.21, 95% CI: 0.07 to 0.60, 4 studies, 326 participants) compared with CM alone. Liu et al. [26] reported that oral Jinpi HM as a monotherapy or an adjunctive therapy to CM showed significantly lower recurrence rate (OR: 0.36, 95% CI: 0.21 to 0.60, 4 studies, 283 participants) compared with CM alone. However, Liu et al. [27] reported that Tripterygium agents as a monotherapy or an adjunctive therapy to CM showed no significant difference in terms of recurrence rate (RR: 0.44, 95% CI: 0.06 to 3.00, 2 studies, 149 participants) compared with CM alone. Ma et al. [24] reported that oral or external HM as an adjunctive therapy to CM showed no significant difference in terms of recurrence rate (OR 0.74, 95% CI 0.36 to 1.53, 3 studies, 251 participants) compared with CM alone. In addition, Wang et al. [28] reported that there was no difference between HM and CM in terms of recurrence rate (RR: 0.57, 95% CI: 0.32 to 1.02, 7 studies, 906 participants) (Table 1).

*(3) Laboratory Results*. The effect of HM on laboratory results was dependent on the outcomes. First, the positive effects of HM on interferon-gamma (IFN-*γ*) and IL-4 have been reported consistently in two reviews. Liu et al. [26] reported that oral Jinpi HM as a monotherapy or an adjunctive therapy to CM showed significantly higher serum IFN-*γ* (MD: 1.75, 95% CI: 1.14 to 2.35, 4 studies, 346 participants) and lower serum IL-4 (MD: −3.15, 95% CI: −4.16 to −2.15, 4 studies, 346 participants). In addition, Liu et al. [27] reported that Tripterygium agents as a monotherapy or an adjunctive therapy to CM showed significantly higher serum IFN-*γ* (SMD: 0.69, 95% CI: 0.37 to 1.01, 1 study, 160 participants) and lower serum IL-4 (SMD: −0.64, 95% CI: −0.96 to −0.33, 1 study, 160 participants). Second, the positive effects of HM on EOS and C-reactive protein (CRP) have been reported. Liu et al. [26] reported that oral Jinpi HM as a monotherapy or an adjunctive therapy to CM showed significantly lower serum EOS (MD: −0.11, 95% CI: −0.20 to −0.02, 5 studies, 410 participants) compared with CM alone. Liu et al. [27] reported that Tripterygium agents as a monotherapy or an adjunctive therapy to CM showed significantly lower serum CRP (SMD: −20.01, 95% CI: −22.64 to −17.39, 1 study, 118 participants) compared with CM alone. Third, HM had no significant effects on IL-2. Liu et al. [27] reported that Tripterygium agents as a monotherapy or an adjunctive therapy to CM showed no significant difference in terms of serum IL-2 (SMD 11.09, 95% CI −13.41 to 35.58, 2 studies, 178 participants) compared with CM alone. Fourth, mixed results have been reported for IgE. Liu et al. [27] reported that Tripterygium agents as a monotherapy or an adjunctive therapy to CM showed significantly lower serum IgE (SMD -0.57, 95% CI −1.11 to −0.03, 1 study, 220 participants) compared with CM alone. In addition, Wang et al. [28] reported that oral or external HM showed significantly lower serum IgE (MD: −119.19, 95% CI: −177.93 to −60.45, 5 studies, 464 participants) compared with CM or placebo. However, Yang et al. [23] reported that oral HM as a monotherapy or an adjunctive therapy to CM showed no significant difference in terms of serum IgE (MD: −67.10, 95% CI: −179.63 to 45.43, 2 studies, 181 participants) compared with CM alone. Liu et al. [26] reported that oral Jinpi HM as a monotherapy or an adjunctive therapy to CM showed no significant difference in terms of serum IgE (MD: −34.92, 95% CI: −86.07 to 16.22, 6 studies, 534 participants) compared with CM alone (Table 1).

#### 3.4.3. Other Comparisons

Shi et al. [25] reported that oral HM, acupuncture, moxibustion, and so on were associated with a decrease in the EASI score (MD: 3.22, 95% CI: 0.41 to 6.03, 2 studies, 50 participants). However, there was no significant difference between groups in terms of TER (RR: 1.10, 95% CI: 0.99 to 1.21, 8 studies, 667 participants), SCORAD score (SMD: 0.89, 95% CI: −0.24 to 2.02, 4 studies, 173 participants), and reduction of symptom scores or index scores (SMD: −0.36, 95% CI: −1.16 to 0.45, 2 studies, 105 participants). However, the review is based on a small number of and low-quality RCTs; therefore, caution must be taken when interpreting our results (Table 1).

### 3.5. Is Oral HM Effective and Safe for Treating AD?

We collected and reanalyzed the original RCTs that matched each comparison from each SR: (1) oral HM versus placebo (4 RCTs), (2) oral HM versus active controls (37 RCTs), and (3) oral HM combined with active controls versus active controls (17 RCTs). We also assessed the quality of evidence using the GRADE approach based on the reanalyzed data on efficacy and safety. The list of the included original RCTs is described in Supplement 2.

#### 3.5.1. HM versus Placebo

Compared with the placebo group, the oral HM group showed significantly lower SCORAD score (MD: −10.65, 95% CI: −16.24 to −5.06, 1 study, 25 participants, low-quality evidence) and higher TER (RR: 9.43, 95% CI: 1.44 to 61.85, 1 study, 25 participants, low-quality evidence). Moreover, there was no significant difference between the groups in terms of the incidence of AEs (RR: 1.23, 95% CI: 0.65 to 2.35, 3 studies, 178 participants, low-quality evidence). The levels of evidence for the results evaluated by the GRADE approach were all “low” because of the presence of RoB in the included RCTs and imprecision of the results (Table 3).

#### 3.5.2. HM versus Active Controls 

Compared with active controls, the oral HM group showed significantly lower SCORAD score (MD: −11.39, 95% CI: −14.21 to −8.57, 17 studies, 1285 participants, low-quality evidence) and higher TER (RR: 1.31, 95% CI: 1.23 to 1.40, 33 studies, 2812 participants, moderate-quality evidence). Moreover, oral HM showed a significantly lower incidence of AEs than active controls (RR: 0.40, 95% CI: 0.26 to 0.64, 19 studies, 1507 participants, low-quality evidence). The levels of evidence for the SCORAD score and the incidence of AEs were “low,” while the level of evidence for TER was “moderate” (Table 3).

#### 3.5.3. HM Combined with Active Controls versus Active Controls Alone

Compared with active controls, oral HM combined with active controls showed significantly lower SCORAD score (MD: −6.05, 95% CI: −7.86 to −4.25, 4 studies, 333 participants, moderate-quality evidence) and higher TER (RR: 1.19, 95% CI: 1.06 to 1.34, 17 studies, 1329 participants, low-quality evidence). There was no significant difference between the groups in terms of the incidence of AEs (RR: 1.21, 95% CI: 0.43 to 3.39, 8 studies, 558 participants, very low-quality evidence). The levels of evidence for SCORAD score, TER, and the incidence of AEs were “moderate,” “low,” and “very low,” respectively (Table 3).

### 3.6. Components of Oral HM for AD

Of the original RCTs, the components of oral HM used in 55 RCTs were analyzed, except for one study that did not provide the components of oral HM used and two studies that did not provide basic prescriptions. As a result, a total of 110 kinds of components including 109 herbs and 1 ingredient of herb, Tripterygium glycosides, were found. The most frequently used herb was *Glycyrrhizae Radix et Rhizoma* (39/55, 70.91%), followed by *Atractylodis Rhizoma Alba* (37/55, 67.27%), *Poria (Hoelen)* (32/55, 58.18%), *Angelicae Gigantis Radix* (22/55, 40.0%), *Dictamni Radicis Cortex* (22/55, 40.0%), *Atractylodis Rhizoma* (19/55, 34.55%), *Saposhnikoviae Radix* (19/55, 34.55%), *Citri Unshius Pericarpium* (18/55, 32.73%), *and Astragali Radix* (16/55, 29.09%). The frequency of the herbs used is shown in Supplement 3.

## 4. Discussion

This overview aimed to summarize and critically evaluate the efficacy and safety of HM on AD, based on current available SRs. Through a comprehensive search in five major medical databases, a total of 9 SRs [7, 21–28] including 6–37 RCTs were included in this overview.

### 4.1. Summary of Evidences

The overview has the advantage of summarizing and analyzing studies that have been excluded from each SR due to various reasons such as differences in research questions. Moreover, we resynthesized the evidence for efficacy and safety of HM on AD by extracting the original RCTs of each SR, to provide updated and more comprehensive evidence. First, we summarized the results from the included SRs. Two reviews [7, 22] evaluated the anti-AD effects of HM compared with the placebo. They found that HM showed significantly better efficacy in terms of TER, symptom scores including itching and sleep symptom, quality of life (QOL), and a dose of topical treatment used compared with placebo. However, all meta-analyses included four or fewer RCTs, and the included RCTs had mostly uncertain or high RoB. Therefore, the reliability of the results could be limited. In addition, HM as a monotherapy and/or an adjunctive therapy to CM showed significantly better clinical outcomes, symptom relief, and results on some laboratory parameters including serum IFN-*γ*, IL-4, EOS, and CRP. In addition, mixed results were found on the recurrence rate and serum IgE. The methodological quality of each SR assessed using the AMSTAR-2 tool was generally low except for one Cochrane review [22]. In particular, only one study [22] reported the review methods prior to the conduct of the review and provided a list of excluded studies. This means that most included reviews might not provide an accurate and comprehensive summary of the included RCTs. Second, we obtained the full texts of 58 original RCTs from each SR to resynthesize the results, especially on efficacy and safety data including the SCORAD score, TER, and the incidence of AEs. As a result, oral HM showed a significantly lower SCORAD score and higher TER than the placebo. In addition, oral HM as a monotherapy or an adjunctive therapy to CM showed lower SCORAD score and higher TER compared with CM alone. Moreover, in terms of the safety profile, oral HM was not significantly different from the placebo and was better than CM. However, the quality of evidence for each finding assessed using the GRADE approach ranged from “moderate” to “very low,” and there was no high-quality evidence provided. We downgraded the quality of evidence because of the high RoB in the included RCTs and the imprecision of the meta-analyzed data. Therefore, limited evidence suggested a positive efficacy of HM on AD, but it is difficult to draw definite conclusions owing to the low methodological quality of each review and poor quality of the evidence.

### 4.2. Clinical Implications

Recently, HM has attracted attention as a promising alternative treatment for AD. According to a cross-sectional survey conducted in the United States in the early 2000s, CIM use in AD patients was very common at about 50% [29]; in a recent population-based study of the 2007 National Health Interview Survey, 47% of pediatric patients with eczema used CIM [30]. However, since abuse of unproven CIM approaches can be a barrier to optimal management of AD [31], evidence-based and rationality-based use of CIM is required. The important findings of this overview are as follows: HM may be used as an adjunctive therapy in AD patients who are receiving conventional treatments and HM may be used as a monotherapy in AD patients who do not want to or cannot apply conventional treatments. This has the clinical significance of presenting a new evidence-based treatment option for patients with this intractable skin disease that severely affects their QOL. In particular, the results of this overview showed that, in the treatment of AD, HM may have immunomodulatory and anti-inflammatory effects, as evidenced by improvements in IFN-*γ*, IL-4, EOS, and CRP. These findings are consistent with those of previous studies, which reported that HM can prevent the development of AD, through complex mechanisms mainly related to inflammation and skin function improvement [32]. Interestingly, in the component analysis of 55 original RCTs using oral HM, the EATM theory indicated that frequently used herbs have tonifying effects or clearing heat-dampness effects. Among them, *Glycyrrhizae Radix et Rhizoma*, *Atractylodis Rhizoma Alba*, *Poria (Hoelen)*, *Angelicae Gigantis Radix*, *Atractylodis Rhizoma*, and *Astragali Radix* are commonly used to tonify *qi* and blood and to treat spleen-stomach dampness and weakness. Meanwhile, *Dictamni Radicis Cortex* is a herb well known for its clearing heat-dampness effect. The frequent use of these herbs is consistent with previous epidemiological studies; for example, the most frequently used prescription for recalcitrant AD treatment in Japan was Hochu-ekki-to, a tonifying qi HM [33], and in Taiwan, Xiao-Feng-San, a clearing heat-dampness HM. Moreover, *Dictamni Radicis Cortex* has been found to be frequently used [34–36]. There are also experimental evidences supporting that these herbs have anti-inflammatory and antiallergic effects, which may help with AD treatment. *Angelicae Gigantis Radix* and the main component (glycyrrhizin) and derivatives (dipotassium glycyrrhizinate) of *Glycyrrhizae Radix et Rhizoma* showed excellent anti-inflammatory effects in the in vitro and in vivo AD models [37–39]. Moreover, these two herbs have shown to reduce inflammation and improve AD symptoms such as itching in the AD model even in topical use [40, 41]. *Atractylodis Rhizoma Alba* and *Poria (Hoelen)* are frequently used herb combinations, which demonstrated anti-allergic and immunomodulatory effects in AD models, respectively [42–44]. *Dictamni Radicis Cortex* is a herb with potent anti-inflammatory and antiallergic effects and is of interest in allergic diseases clinically and experimentally [36, 45, 46]. However, the safety profiles of HM as an adjuvant therapy in terms of herb-drug interaction need further studies. Although some studies have reported that the combination of CM such as oral antihistamines and HM in AD treatment was effective and safe [47], the interactions between herbs and CMs used in AD were largely understudied. For example, in the case of systemic immunosuppressive agents such as cyclosporine for severe AD, the administration of HM metabolized by the cytochrome P450 (CYP) system may affect its efficacy by changing the bioavailability of the drug. Some herbs including St. John's wort, ginger, liquorice, Scutellariae radix, and quercetin have been known to decrease the bioavailability of cyclosporine, while other herbs including grapefruit juice, cannabidiol, chamomile, resveratrol, *Serenoa repens*, *Echinacea*, and berberine have been known to increase the bioavailability [48]. This herb-drug interaction is an important issue in current clinical settings, especially in terms of CIM, and should be taken into account when establishing an optimal holistic treatment strategy [49]. The results of meta-analysis suggest that there was no difference in the incidence of AEs between HM combined with CM and CM alone, but the level of evidence evaluated by GRADE was very low. This finding suggests that studies evaluating their interaction should be conducted, given the priority interaction between the drugs currently used and the HMs frequently used in treating AD, as proposed in this overview. Additionally, these studies should be particularly important in terms of immunomodulation and common drug metabolic systems.

### 4.3. Limitations and Suggestions for Future Studies

Our overview has some limitations. First, all included SRs as well as most of the original RCTs were published in China. This may seem reasonable because HM is primarily used in China. However, this can still lead to reporting biases and barriers to generalizing HM in other countries. Second, most of the reviews included had low methodological quality. Although our overview included a high-quality SR, such as the Cochrane review, the overall quality of included SRs appeared to be low based on the AMSTAR-2 assessment results. Literature review, including the SR, can be considered as a kind of secondary analysis of the original articles. Therefore, it is necessary to exclude the bias of the researcher as much as possible. This may include preprotocol registration and predefined analysis methods. There is also a need to use a multifaceted approach to explain significant heterogeneity, with proper methods. Like most SRs included in this overview, the interventions used in many SRs of HM have a great variety in their composition. This is thought to be a feature of EATM that uses different treatments for the same disease or vice versa, which has led to heterogeneity in many SRs. Recently, however, as a way to solve this problem, analyses limited to HM with specific therapeutic properties have also been conducted, like the review by Liu et al. [26]. In addition, as in Liu et al. [27], analyses of HM focused on a specific herb can be conducted. In particular, in the former case, the pattern identification system of EATM can be used, and the contents of the previously published CPG may be utilized. For example, the CPG of HM treatment for AD, published in 2014 in Korea, classified the patterns of AD as follows [10]: dampness-heat (濕熱), fetal heat (胎熱), syndrome of spleen deficiency with wind-dryness (脾虛風燥), wind-dampness skin syndrome (風濕蘊膚), syndrome of damp-heat gluing each other (濕熱互結), spleen deficient with dampness (脾虛濕蘊), and syndrome of blood deficiency and wind-dryness (血虛風燥). Some HMs corresponding to each pattern may have common therapeutic characteristics, and it is thought that it can be integrated into intervention condition or separated by subgroup analysis in future SR. Through these studies, it is necessary to conduct research for the standardization of HM for AD and reflect it in the updated CPG. In addition, comparative effectiveness between various HMs should be explored using a network meta-analysis in future studies. Third, the overall quality of the original RCTs included was also poor. In addition, the quality of evidence assessed using GRADE ranged from very low to moderate. For HM, a double-blind and placebo-controlled RCT is essential to confirm its efficacy and safety. However, the number of such studies included in our reanalysis was very limited. This suggests that high-quality, large, rigorous RCTs should be performed in order for HM to be recognized as a solid treatment for AD. Fourth, there was a lack of strict AE reporting in the original RCTs included. As described above, the generalization of HM in medical choice requires the establishment of a clear basis for HM safety profile, particularly the herb-drug interaction. Given that many AD patients already use CIM therapies including HM [29, 30], evaluations of the herb-drug interaction between the drugs currently used and the HMs frequently used in treating AD are highly needed. Furthermore, for HM to be considered a policy for the treatment of AD, it is necessary to conduct a study evaluating the cost-effectiveness of HM for AD. Finally, the therapeutic mechanism of HM for AD needs to be further elucidated. For example, the gut-skin axis has been found to play an important role in the pathology of AD [50], and given that the impact on the microbiome is one of the complex therapeutic mechanisms of HM [51], anti-AD effects of HM via the gut-skin axis should be further elucidated.

Despite these limitations, to the best of our knowledge, this is the first overview to systematically summarize and evaluate the efficacy and safety HM on AD from current SRs. In addition, we reextracted and resynthesized the information related to efficacy and safety from the RCTs included in each SR to provide more updated and comprehensive information. This overview provided an evidence-based assessment and object summary on the efficacy and safety of HM on AD. We believe that this overview will facilitate the evidence-based use of HM as a way to optimize tailored care for healthcare practitioners who treat AD and policymakers. The results of this overview will also provide researchers with insights on the urgent research challenges in this area.

## 5. Conclusion

In conclusion, most of the included reviews and resynthesized results suggested that HM might be effective for the treatment of AD. In particular, HM generally appears to have immunomodulatory and anti-inflammatory effects. However, the methodological quality of the included reviews and original RCTs and the quality of evidence for the main findings were generally low. In addition, since most included RCTs were not strict in reporting AE, it might not sufficiently report the safety issues of HM and the herb-drug interactions. Therefore, further rigorous, well-designed, high-quality SRs and RCTs are needed to draw a firm conclusion.

## Figures and Tables

**Table 1:** Characteristics of included systematic reviews.

First author (year)	First author's country	Studies (sample size)	Search duration	Control intervention	Main results (meta-analysis)	Key conclusion
Gong (2009) [21]	China	11 RCTs (1,479)	Inception-2007.12.	Oral antihistamines	(I) Oral HM vs. oral antihistamines	Meta-analysis showed that HM is effective in treating AD, and its curative effect is superior to antihistamine. The conclusion of this study is credible, suggesting that HM treatment of AD has positive prospects.
					(1) TER^*∗*^	
					11 RCTs, *n* = 1479; OR 5.64, 95% CI 4.07 to 7.81, *I*^2^ = 0%	
					(2) Recurrence rate+	
					11 RCTs, *n* = 1436; OR 0.38, 95% CI 0.29 to 0.49, *I*^2^ = 0%	

Gu (2013) [22]	China	28 RCTs (2,306)	Inception-2012.9.	Placebo, no treatment, or active controls	(I) Oral or external HM vs. placebo	We could not find conclusive evidence that oral or external HM could reduce the severity of eczema in children or adults. We assessed most of the studies as high risk of bias, particularly in blinding of participants and personnel, and there was substantial inconsistency between studies, so any positive effect of HM must be interpreted with caution.
					(1) TER^*∗*^	
					2 RCTs, *n* = 85; RR 2.09, 95% CI 1.32 to 3.32, *I*^2^ = 0%	
					(2) Itching VAS+	
					2 RCTs, *n* = 94; SMD −1.53, 95% CI −2.64 to −0.41, *I*^2^ = 74%	
					(3) Overall severity+	
					4 RCTs, *n* = 239; SMD −0.88, 95% CI −1.67 to −0.09, *I*^2^ = 87%	
					(4) CDLQI score+	
					1 RCT, *n* = 85; MD −2.50, 95% CI −4.77 to −0.23	
					(II) Oral or external HM vs. CM	
					(1) TER^*∗*^	
					21 RCTs, *n* = 1868; RR 1.43, 95% CI 1.27 to 1.61, *I*^2^ = 65%	
					(2) Itching VAS+	
					7 RCTs, *n* = 465; SMD −0.83, 95% CI −1.43 to −0.22, *I*^2^ = 89%	
					(3) Overall severity+	
					15 RCTs, *n* = 1062; SMD −0.97, 95% CI −1.23 to −0.71, *I*^2^ = 74%	
					(III) Oral or external HM vs. oral HM	
					(1) TER^*∗*^	
					1 RCT, *n* = 20; RR 1.13, 95% CI 0.78 to 1.63	
					2) Itching VAS+	
					1 RCT, *n* = 23; MD −1.05, 95% CI −1.75 to −0.35	
					(3) Skin lesion score+	
					1 RCT, *n* = 23; MD −1.59, 95% CI −2.92 to −0.26	
					(4) Overall severity+	
					1 RCT, n=20; MD -3.43, 95% CI -7.01 to 0.15	
					5) CDLQI score+	
					1 RCT, *n* = 20; MD 0.90, 95% CI −2.89 to 4.69	

Tan (2013) [7]	China	6 RCTs (432)	Inception-2011.	Placebo, no treatment, or active controls	(I) Oral HM vs. placebo	HM significantly improved symptom severity of AD and it was reported as well-tolerated. The overall risk of bias assessment found that the quality of studies was poor; therefore, the results from the meta-analysis have to be interpreted with caution.
					(1) Erythema score+	
					3 RCTs, *n* = 245; SMD −0.76, 95% CI −1.05 to −0.47, the value of *I*^2^ was not presented.	
					(2) Surface damage score+	
					3 RCTs, *n* = 245; SMD −1.08, 95% CI −1.59 to −0.56, the value of *I*^2^ was not presented.	
					(3) Itching score+	
					1 RCT, *n* = 71; MD −1.10, 95% CI −1.59 to −0.61	
					(4) Sleep score+	
					1 RCT, *n* = 71; MD −0.80, 95% CI −1.12 to −0.48	
					(5) CDLQI score+	
					1 RCT, *n* = 85; MD −2.50, 95% CI −4.77 to −0.23	
					(6) Dose of topical treatment used+	
					1 RCT, *n* = 91; MD −24.50, 95% CI −27.92 to −21.08	
					(II) Oral HM + CM vs. CM	
					(1) Overall severity score+	
					1 RCT, *n* = 98; MD −2.56, 95% CI −3.46 to −1.66	

Yang (2016) [23]	China	13 RCTs (1,232)	2000.01.01-2015.10.31	CM	(I) Oral HM (or oral HM + CM) vs. CM	The current clinical evidence showed that HM treatment for AD has better clinical effect. Due to poor methodological quality of existing trials, the future need more high quality, large-sample RCTs to get more reliable clinical conclusions.
					(1) TER^*∗*^	
					13 RCTs, *n* = 1232; OR 4.86, 95% CI 3.44 to 6.87, *I*^2^ = 0%	
					(2) SCORAD score+	
					4 RCTs, *n* = 390; MD −15.51, 95% CI −20.15 to −10.87, *I*^2^ = 73%	
					(3) Serum IgE+	
					2 RCTs, *n* = 181; MD −67.10, 95% CI −179.63 to 45.43, *I*^2^ = 87%	
					(4) Recurrence rate+	
					4 RCTs, *n* = 326; OR 0.21, 95% CI 0.07 to 0.60, *I*^2^ = 58%	

Ma (2017) [24]	China	14 RCTs (976)	Inception-2016.9.	CM	(I) Oral or external HM + CM vs. CM	The present study suggests that combination of HM and CM has curative effect with lower incidence of adverse reactions in the treatment of AD. The search results are limited to domestic literature, the clinical evidence level is low, and there is a lack of high-quality, standardized RCT. Further RCTs are required to confirm it.
					(1) Cure rate^*∗*^	
					11 RCTs, *n* = 757; OR 2.94, 95% CI 2.08 to 4.16, *I*^2^ = 0%	
					(2) TER^*∗*^	
					12 RCTs, *n* = 733; OR 4.86, 95% CI 3.13 to 7.56, *I*^2^ = 0%	
					(3) Recurrence rate+	
					3 RCTs, *n* = 251; OR 0.74, 95% CI 0.36 to 1.53, *I*^2^ = 0%	

Shi (2017) [25]	China	24 RCTs (1,618)	Inception-2016.12.	Placebo or CM	(I) Oral HM, acupuncture, moxibustion, etc. vs. placebo or CM	We need to make conclusion cautiously for the efficacy and safety of HM and related treatment on AD. Articles having good quality based on the Cochrane Collaboration's risk of bias tool were included ensuring the results trustworthy.
					(1) TER^*∗*^	
					8 RCTs, *n* = 667; RR 1.10, 95% CI 0.99 to 1.21, *I*^2^ = 65%	
					(2) SCORAD score+	
					4 RCTs, *n* = 173; SMD 0.89, 95% CI −0.24 to 2.02, *I*^2^ = 86%	
					(3) decrease of EASI score^*∗*^	
					2 RCTs, *n* = 50; MD 3.22, 95% CI 0.41 to 6.03, *I*^2^ = 0%	
					(4) decrease of SSRI score^*∗*^	
					2 RCTs, *n* = 105; SMD −0.36, 95% CI −1.16 to 0.45, *I*^2^ = 76%	

Liu 2018 [26]	China	37 RCTs (2,973)	Inception-2017.12.	CM	(1) Oral Jinpi HM (or oral Jinpi HM + CM) vs. CM	Studies have shown that HM Jianpi therapy had significantly higher clinical efficacy than CM in the treatment of AD. Due to the publication bias and low quality of included RCTs in this study, more multicenter, high quality, large-sample, randomized double-blind controlled trials are needed to further demonstrate the conclusion.
					(1) TER^*∗*^	
					30 RCTs, *n* = 2333; OR 4.05, 95% CI 3.27 to 5.03, *I*^2^ = 0%	
					(2) SCORAD score+	
					15 RCTs, *n* = 1282; MD −9.82, 95% CI −13.31 to −6.33, *I*^2^ = 90%	
					(3) EASI score+	
					3 RCTs, *n* = 232; MD −2.80, 95% CI −3.54 to −2.07, *I*^2^ = 0%	
					(4) Itching VAS+	
					7 RCTs, *n* = 448; MD −0.79, 95% CI −1.10 to −0.47, *I*^2^ = 24%	
					(5) Serum IgE+	
					6 RCTs, *n* = 534; MD −34.92, 95% CI −86.07 to 16.22, *I*^2^ = 97%	
					(6) Serum IFN-*γ*^*∗*^	
					4 RCTs, *n* = 346; MD 1.75, 95% CI 1.14 to 2.35, *I*^2^ = 0%	
					(7) Serum IL-4+	
					4 RCTs, *n* = 346; MD −3.15, 95% CI −4.16 to −2.15, *I*^2^ = 75%	
					(8) Serum EOS+	
					5 RCTs, *n* = 410; MD −0.11, 95% CI −0.20 to −0.02, *I*^2^ = 0%	
					(9) Recurrence rate+	
					4 RCTs, *n* = 283; OR 0.36, 95% CI 0.21 to 0.60, *I*^2^ = 0%	
					(II) Oral Jinpi HM vs. CM	
					(1) TER^*∗*^	
					21 RCTs, *n* = 1355; OR 4.81, 95% CI 3.63 to 6.36, *I*^2^ = 0%	
					(III) Oral Jinpi HM + CM vs. CM	
					(1) TER^*∗*^	
					9 RCTs, *n* = 832; OR 2.94, 95% CI 2.11 to 4.11, *I*^2^ = 0%	

Liu (2019) [27]	China	13 RCTs (1385)	Inception-2018.10.2.	CM	(I) Tripterygium agents (or Tripterygium agents + CM) vs. CM	Tripterygium agents appear to be effective when treating patients with atopic eczema, but with apparent side effects. It cannot be concluded that Tripterygium agents can be generally used for eczema in the clinic because of the small sample size. Further multi-center studies with large samples, and high-quality should be conducted to clarify the efficacy and safety of Tripterygium agents for treating eczema.
					(1) TER^*∗*^	
					13 RCTs, *n* = 1361; RR 1.59, 95% CI 1.26 to 2.00, *I*^2^ = 93%	
					(2) Serum IL-2^*∗*^	
					2 RCTs, *n* = 178; SMD 11.09, 95% CI −13.41 to 35.58, *I*^2^ = 99%	
					(3) Serum IL-4+	
					1 RCT, *n* = 160; SMD −0.64, 95% CI −0.96 to −0.33	
					(4) Serum IFN-*γ*^*∗*^	
					1 RCT, *n* = 160; SMD 0.69, 95% CI 0.37 to 1.01	
					(5) Serum CRP+	
					1 RCT, *n* = 118; SMD −20.01, 95% CI −22.64 to −17.39	
					(6) Serum IgE+	
					1 RCT, *n* = 220; SMD −0.57, 95% CI −1.11 to −0.03	
					(7) Recurrence rate+	
					2 RCTs, *n* = 149; RR 0.44, 95% CI 0.06 to 3.00, *I*^2^ = 89%	
					(II) Tripterygium agents vs. CM	
					(1) TER^*∗*^	
					4 RCTs, *n* = 367; RR 1.19, 95% CI 0.96 to 1.48, *I*^2^ = 85%	
					(III) Tripterygium agents + CM vs. CM	
					(1) TER^*∗*^	
					9 RCTs, *n* = 994; RR 1.78, 95% CI 1.40 to 2.25, *I*^2^ = 84%	

Wang (2019) [28]	China	19 RCTs (2178)	1980.1.1.-2019.3.31.	Placebo or active controls	(I) Oral or external HM vs. CM	HM have certain advantages in treating atopic dermatitis and have relatively lower incidents of adverse reaction.
					(1) Cure rate^*∗*^	
					16 RCTs, *n* = 1727; RR 1.79, 95% CI 1.35 to 2.39, *I*^2^ = 60%	
					(2) TER^*∗*^	
					16 RCTs, *n* = 1727; RR 1.19, 95% CI 1.08 to 1.31, *I*^2^ = 90%	
					(3) SCORAD score+	
					4 RCTs, *n* = 292; MD −14.67, 95% CI −19.52 to −9.82, *I*^2^ = 77%	
					(4) Recurrence rate+	
					7 RCTs, *n* = 906; RR 0.57, 95% CI 0.32 to 1.02, *I*^2^ = 76%	
					(5) Adverse events rate+	
					14 RCTs, *n* = 1735; RR 0.49, 95% CI 0.36 to 0.66, *I*^2^ = 42%	
					(II) Oral or external HM vs. CM or Placebo	
					(1) Serum IgE+	
					5 RCTs, *n* = 464; MD −119.19, 95% CI −177.93 to −60.45, *I*^2^ = 0%	

^*∗*^Higher score indicates better results, ^+^lower score indicates better results. AD, atopic dermatitis; CDLQI, children's dermatology life quality index; CI, confidence interval; CM, conventional medication; CRP, C-reactive protein; EASI, eczema area and severity index; EOS, eosinophil count; HM, herbal medicine; IFN, inteferon; IL, interleukin; IgE, immunoglobulin E; MD, mean difference; OR, odds ratio; RCT, randomized controlled trial; RR, risk ratio; SCORAD, scoring atopic dermatitis; SMD, standardized mean difference; SSRI, symptom score reducing index; TER, total effective rate; VAS, visual analogue scale.

**Table 2:** Methodological quality assessment of the included reviews using the AMSTAR-2 tool.

Included studies	Q1	Q2	Q3	Q4	Q5	Q6	Q7	Q8	Q9	Q10	Q11	Q12	Q13	Q14	Q15	Q16	Overall quality
Gong [21]	Yes	No	Yes	No	Yes	Yes	No	No	No	No	Yes	Yes	No	Yes	Yes	Yes	Critically low
Gu et al. [22]	Yes	Yes	Yes	Yes	Yes	Yes	Yes	Yes	Yes	Yes	Yes	Yes	Yes	Yes	Yes	Yes	High
Tan et al. [7]	Yes	No	Yes	Partial yes	No	Yes	No	Yes	Yes	No	Yes	No	Yes	Yes	Yes	Yes	Critically low
Yang et al. [23]	Yes	No	Yes	No	Yes	Yes	No	Partial yes	No	No	No	No	Yes	No	Yes	Yes	Critically low
Ma et al. [24]	Yes	No	Yes	Partial yes	No	Yes	No	Partial yes	No	No	Yes	No	Yes	Yes	Yes	Yes	Critically low
Shi et al. [25]	Yes	No	Yes	Partial yes	Yes	Yes	No	Yes	Yes	No	Yes	Yes	Yes	Yes	No	Yes	Critically low
Liu et al. [26]	Yes	No	Yes	Partial yes	No	No	No	Partial yes	Yes	No	No	No	Yes	No	Yes	Yes	Critically low
Liu et al. [27]	Yes	Yes	Yes	Yes	Yes	Yes	No	Yes	Yes	No	Yes	No	Yes	Yes	Yes	Yes	Low
Wang et al. [28]	Yes	No	Yes	Partial yes	Yes	Yes	No	Partial yes	Yes	No	Yes	No	Yes	Yes	Yes	Yes	Critically low

Q1: did the research questions and inclusion criteria for the review include the components of PICO? Q2: did the report of the review contain an explicit statement that the review methods were established prior to the conduct of the review and did the report justify any significant deviations from the protocol? Q3: did the review authors explain their selection of the study designs for inclusion in the review? Q4: did the review authors use a comprehensive literature search strategy? Q5: did the review authors perform study selection in duplicate? Q6: did the review authors perform data extraction in duplicate? Q7: did the review authors provide a list of excluded studies and justify the exclusions? Q8: did the review authors describe the included studies in adequate detail? Q9: did the review authors use a satisfactory technique for assessing the risk of bias (RoB) in individual studies that were included in the review? Q10: did the review authors report on the sources of funding for the studies included in the review? Q11: if meta-analysis was performed, did the review authors use appropriate methods for statistical combination of results? Q12: if meta-analysis was performed, did the review authors assess the potential impact of RoB in individual studies on the results of the meta-analysis or other evidence synthesis? Q13: did the review authors account for RoB in individual studies when interpreting/discussing the results of the review? Q14: did the review authors provide a satisfactory explanation for, and discussion of, any heterogeneity observed in the results of the review? Q15: if they performed quantitative synthesis, did the review authors carry out an adequate investigation of publication bias (small study bias) and discuss its likely impact on the results of the review? Q16: did the review authors report any potential sources of conflicts of interest, including any funding they received for conducting the review?

**Table 3:** Quality of evidence for the main findings.

Outcome	No. of participants (RCTs)	Quality of evidence (GRADE)	Relative risk (95% CI)	Anticipated absolute effects (95% CI)		Comments
				Risk with control intervention	Risk with treatment intervention	
*HM versus placebo*						
SCORAD score	25 (1 RCT)	⊕⊕○○ LOW	—	—	MD 10.65 lower [-16.24, -5.06]	Risk of bias (−1) Imprecision (−1)
TER	25 (1 RCT)	⊕⊕○○ LOW	RR 9.43 [1.44, 61.85]	91 per 1,000	857 per 1,000 [131, 1,091]	Risk of bias (−1) Imprecision (−1)
Adverse events rate	178 (3 RCTs)	⊕⊕○○ LOW	RR 1.23 [0.65, 2.35]	154 per 1,000	189 per 1,000 [100, 362]	Risk of bias (−1) Imprecision (−1)

*HM versus active controls*						
SCORAD score	1,285 (17 RCTs)	⊕⊕○○ LOW	—	—	MD 11.39 lower [-14.21, -8.57]	Risk of bias (−1) Inconsistency (−1)
TER	2,812 (33 RCTs)	⊕⊕⊕○ MODERATE	RR 1.31 [1.23, 1.40]	703 per 1,000	921 per 1,000 [864, 984]	Risk of bias (−1)
Adverse events rate	1,507 (19 RCTs)	⊕⊕○○ LOW	RR 0.40 [0.26, 0.64]	98 per 1,000	39 per 1,000 [25, 63]	Risk of bias (−1) Imprecision (−1)

*HM combined with active controls versus active controls alone*						
SCORAD score	333 (4 RCTs)	⊕⊕⊕○ MODERATE	—	—	MD 6.05 lower [-7.86, -4.25]	Risk of bias (−1)
TER	1,329 (17 RCTs)	⊕⊕○○ LOW	RR 1.19 [1.06, 1.34]	797 per 1,000	948 per 1,000 [845, 1,068]	Risk of bias (−1) Publication bias (−1)
Adverse events rate	558 (8 RCTs)	⊕○○○ VERY LOW	RR 1.21 [0.43, 3.39]	78 per 1,000	94 per 1,000 [33, 265]	Risk of bias (−1) Inconsistency (−1) Imprecision (−1)

CI, confidence interval; GRADE, grading of recommendations assessment, development, and evaluation; HM, herbal medicine; MD, mean difference; RCT, randomized controlled trial; RR, risk ratio; SCORAD, scoring atopic dermatitis; TER, total effective rate.

## Supplementary Materials

Supplementary Materials Supplement 1: search strategies for each database. Supplement 2: original randomized controlled trials reanalyzed. Supplement 3: frequency of components of oral HM in original randomized controlled trials reanalyzed.
