# Supplementary material for: Effectiveness and Safety of Herbal Medicine for Atopic Dermatitis: An Overview of Systematic Reviews
Source: Evid Based Complement Alternat Med. 2020 Jul 17;2020:4140692. doi: 10.1155/2020/4140692 (PMC7382724; doi:10.1155/2020/4140692)
Supplement: Supplementary Materials — Supplement 1: search strategies for each database. Supplement 2: original randomized controlled trials reanalyzed. Supplement 3: frequency of components of oral HM in original randomized controlled trials reanalyzed. [file 4140692.f1.docx]

**Supplement 1**. Search strategies for each database

***Medline (via PubMed)***

(Dermatitis, Atopic[MeSH] OR atopic[tiab]) AND (Drugs, Chinese Herbal[MeSH] OR Medicine, East Asian Traditional[MeSH] OR Herbal Medicine[MeSH] OR herbal[tiab])

***Cochrane library***

#1. MeSH descriptor: [Dermatitis, Atopic] explode all trees

#2. (atopic):ti,ab,kw

#3. MeSH descriptor: [Drugs, Chinese Herbal] explode all trees

#4. MeSH descriptor: [Medicine, East Asian Traditional] explode all trees

#5. MeSH descriptor: [Herbal Medicine] explode all trees

#6. (herbal):ti,ab,kw

#7. (#1 OR #2) AND (#3 OR #4 OR #5 OR #6)

***EMBASE (via Elsevier)***

#1. 'atopic dermatitis'/exp OR 'atopic dermatitis'

#2. 'oriental medicine'/exp OR 'oriental medicine'

#3. 'chinese medicine'/exp OR 'chinese medicine'

#4. ‘korean medicine’/exp OR ‘korean medicine’

#5. 'kampo medicine (drug)'/exp OR 'kampo medicine (drug)'

#6. 'traditional medicine'/exp OR 'traditional medicine'

#7. 'chinese drug'/exp OR 'chinese drug'

#8. 'chinese herb'/exp OR 'chinese herb'

#9. 'herbal medicine'/exp OR 'herbal medicine'

#10. #1 AND (#2 OR #3 OR #4 OR #5 OR #6 OR #7 OR #8 OR #9)

***CNKI [subject]***

异位性皮炎 OR 异位性湿疹 OR 特应性皮炎 OR 特异性湿疹

中医药 OR 中药 OR 中医

OASIS

아토피피부염 AND 한약

**Supplement 2**. Original randomized controlled trials re-analyzed

**Comparison I: Oral herbal medicine versus placebo** [1-4]

**Comparison II: Oral herbal medicine versus active controls** [5-41]

**Comparison III: Oral herbal medicine + active controls versus active controls** [42-58]

[1] K.L. Hon, T.F. Leung, P.C. Ng, M.C. Lam, W.Y. Kam, K.Y. Wong, K.C. Lee, Y.T. Sung, K.F. Cheng, T.F. Fok, K.P. Fung, P.C. Leung, Efficacy and tolerability of a Chinese herbal medicine concoction for treatment of atopic dermatitis: a randomized, double-blind, placebo-controlled study, The British journal of dermatology 157(2) (2007) 357-63.

[2] 孙晓冬, 健脾渗湿颗粒治疗特应性皮炎的疗效评价及其对复发的影响, 中国中西医结合皮肤性病学杂志 (06) (2009).

[3] H. Kobayashi, M. Ishii, S. Takeuchi, Y. Tanaka, T. Shintani, A. Yamatodani, T. Kusunoki, M. Furue, Efficacy and Safety of a Traditional Herbal Medicine, Hochu-ekki-to in the Long-term Management of Kikyo (Delicate Constitution) Patients with Atopic Dermatitis: A 6-month, Multicenter, Double-blind, Randomized, Placebo-controlled Study, Evidence-based complementary and alternative medicine : eCAM 7(3) (2010) 367-73.

[4] H.M. Cheng, L.C. Chiang, Y.M. Jan, G.W. Chen, T.C. Li, The efficacy and safety of a Chinese herbal product (Xiao-Feng-San) for the treatment of refractory atopic dermatitis: a randomized, double-blind, placebo-controlled trial, International archives of allergy and immunology 155(2) (2011) 141-8.

[5] 余土根, 朱金土, 许家鸾, 庄亦仁, 沈孛, 吴蓓玲, 何慧英, 马丽俐, 曹毅, 皮炎消净饮Ⅱ号治疗异位性皮炎的临床和实验研究, 中医杂志 (03) (1999) 165-167.

[6] 朱金土, 余土根, 曹毅, 马丽俐, 皮炎消净饮1号治疗湿热型异位性皮炎的临床研究, 浙江中西医结合杂志 (06) (2003) 16-17+23.

[7] 杨瑛, 孙继兰, 王麦娣, 赵天群, 王玥, 杨玉峰, 健脾止痒颗粒治疗特应性皮炎临床观察, 中国皮肤性病学杂志 (12) (2005) 755-756.

[8] 张青松, 健脾化湿方治疗儿童特应性皮炎的临床研究, 2005.

[9] 麻林玖, 梁红梅, 加味启脾丸颗粒治疗儿童特应性皮炎疗效观察, 中国皮肤性病学杂志 (11) (2006) 698.

[10] 杨瑛, 王益平, 杨玉峰, 郭志武, 张尚斌, 健脾止痒颗粒对特应性皮炎患者血清IgE水平的影响, 中医药学刊 (03) (2006) 472-473.

[11] 金培志, 叶秋华, 沈明, 健脾止痒颗粒治疗特应性皮炎32例疗效观察, 河南中医 (12) (2007).

[12] 刘君丽, 马景禄, 健脾消风汤治疗特应性皮炎疗效观察, 中国实用医药 (28) (2008) 99-100.

[13] 杨雪松, 叶建州, 李钦, 健脾养血祛风法治疗特应性皮炎临床疗效及对皮肤屏障功能的影响, 云南中医学院学报 (03) (2009).

[14] 张晓杰, 宋晓莉, 小儿化湿汤治疗小儿湿疹的临床疗效观察, 世界中医药学会联合会皮肤科专业委员会成立大会暨首届国际中医、中西医结合皮肤病学术研讨会论文集, 广州, 2009, pp. 297-299.

[15] 曹珉, 术苓健脾化湿方治疗特应性皮炎的临床研究, 2009.

[16] 龚小红, 匡琳, 刘翔, 凉血消风方治疗成人异位性皮炎的临床观察, 湖南中医药大学学报 (03) (2010).

[17] 瞿平元, 蔡正良, 李亚琴, 刘辉, 胡明芝, 清热祛风汤治疗特应性皮炎30例临床疗效观察, 甘肃医药 29(06) (2010) 646-648.

[18] 罗凤娇, 消风散加减治疗异位性皮炎之疗效评估, 2010.

[19] 赖新平, 雷公藤多甙片治疗异位性皮炎的临床研究, 海南医学院学报 16(08) (2010) 1060-1062.

[20] 赵喆, 劳淑冰, 夏正雄, 吴晓霞, 健脾消导汤治疗儿童特应性皮炎58例疗效观察, 新中医 42(08) (2010) 70-71.

[21] 肖卫棉, 淮莲饮治疗特应性皮炎临床观察, 广州中医药大学, 2010.

[22] 李勇, 廖梦怡, 李东海, 朱其杰, 健脾养阴法治疗特应性皮炎临床研究, 新中医 43(02) (2011) 92-93.

[23] 林素财, 健脾止痒汤治疗特应性皮炎临床疗效观察, 广州中医药大学, 2011.

[24] 薛素琴, 谭金华, 生血润肤饮加减治疗儿童特应性皮炎临床观察, 新中医 (02) (2011).

[25] 张池金, 滋阴清热法治疗特应性皮炎30例临床观察, 中医药导报 (08) (2011).

[26] 莫秀梅, 培土清心法治疗特应性皮炎的多维临床疗效评价研究, 广州中医药大学, 2012.

[27] 郑永平, 谢荣标, 陈高飞, 张玲, 补脾祛风方治疗特应性皮炎临床疗效观察, 光明中医 27(07) (2012) 1365-1366.

[28] 秦悦思, 健脾安神法治疗特应性皮炎临床疗效观察, 成都中医药大学, 2012.

[29] 才吉甫, 向红芬, 运用中药治疗儿童特应性皮炎在焉耆盆地的观察, 中国伤残医学 21(10) (2013) 257.

[30] 彭勇, 李斌, 李锋, 王英杰, 武宗琴, 柴维汉, 健脾祛风方治疗特应性皮炎95例临床观察, 上海中医药大学学报 27(03) (2013) 45-47.

[31] 胡太平, 马骏, 严张仁, 益肺健脾法治疗肺脾气虚型儿童期特异性皮炎的临床研究, 现代诊断与治疗 24(15) (2013) 3422-3423.

[32] 傅伟兰, 中医药治疗儿童特应性皮炎的疗效观察, 中外医学研究 12(18) (2014) 12-14.

[33] 王俊志, 西旺, 佟晓辉, 刘畅, 解毒润肤汤治疗特应性皮炎(湿热证)的临床观察, 中医药信息 31(05) (2014) 111-113.

[34] 王海波, 刘海春, 王振清, 健脾安神中药治疗特应性皮炎, 吉林中医药 34(12) (2014) 1255-1257.

[35] 赵廷元, 于小峰, 谢彦, 健脾化湿法治疗儿童特应性皮炎的研究, 中医临床研究 6(15) (2014) 65-67.

[36] 周爱妍, 姬爱华, 史传奎, 高蕾, 健脾化湿法治疗儿童特应性皮炎30例, 西部中医药 27(08) (2014) 73-74.

[37] J. Liu, X. Mo, D. Wu, A. Ou, S. Xue, C. Liu, H. Li, Z. Wen, D. Chen, Efficacy of a Chinese herbal medicine for the treatment of atopic dermatitis: a randomised controlled study, Complementary therapies in medicine 23(5) (2015) 644-51.

[38] 厉秀玲, 用公藤多甙治疗异位性皮炎的效果及对相关指标的影响, 当代医药论丛 (10) (2015) 277-278.

[39] 宋飞妮, 健脾养血汤治疗特应性皮炎临床观察, 深圳中西医结合杂志 25(22) (2015) 51-52.

[40] 欧阳政洁, 清热利湿宣肺汤治疗湿热蕴结型儿童期特异性皮炎30例总结, 湖南中医杂志 3(33) (2017).

[41] 张琳, 润燥祛风汤治疗特应性皮炎的临床观察, 中国实用医药 29(13) (2018).

[42] 朱春友, 中西医结合治疗异位性皮炎疗效观察, 现代医药卫生 (05) (2001) 368.

[43] 周智敏, 中西医结合治疗异位性皮炎41例临床研究, 湖南中医药导报 (03) (2003) 34-35.

[44] 黄咏菁, 陈达灿, 莫秀梅, 健脾渗湿冲剂治疗儿童异位性皮炎脾虚证的临床观察, 陕西中医 (05) (2004) 396-398.

[45] 张玉环, 陈保疆, 中西医结合治疗特应性皮炎的临床研究, 第2届中国中医药发展大会论文集, 北京, 2005, pp. 594-597.

[46] 欧柏生, 刘卫兵, 王建民, 四弯风汤联合西药治疗特应性皮炎34例, 中国民间疗法 (01) (2006) 8-9.

[47] 李菲, 罗文辉, 中西医结合治疗特应性皮炎44例总结, 湖南中医杂志 (04) (2006) 20-21.

[48] 林海桂, 健脾利湿汤治疗儿童异位性皮炎136例, 浙江中医杂志 (07) (2006) 392.

[49] 刘源, 叶秋华, 陈加媛, 贺勤, 王亚美, 魏凯峰, 陆平成, 中西医结合治疗儿童特应性皮炎临床研究, 南京中医药大学学报 (02) (2007) 93-95.

[50] 史永俭, 张春敏, 马冬梅, 刘瑛, 张冬云, 张春红, 魏国, 孟丽亚, 中西医结合治疗特应性皮炎的临床观察, 中国中西医结合杂志 (08) (2008) 686-688.

[51] 陈保疆, 张玉环, 健脾利湿汤治疗异位性皮炎的疗效观察与相关实验研究, 吉林中医药 31(04) (2011) 335-336.

[52] 周琳, 滋阴健脾冲剂治疗特应性皮炎的疗效观察, 湖北中医药大学, 2012.

[53] 陈保疆, 养血健脾润肤法治疗特应性皮炎临床疗效观察及对患者血清白细胞介素18影响, 中国中西医结合皮肤性病学杂志 13(05) (2014) 283-285.

[54] 董心亚, 周健, 健脾化湿汤联合丁酸氢化可的松乳膏治疗特应性皮炎的效果, 广东医学 36(22) (2015) 3540-3542.

[55] 雷小娟, 健脾化湿汤联合丁酸氢化可的松乳膏治疗特应性皮炎的效果, 中外女性健康研究 (19) (2016) 21+24.

[56] 徐岳清, 健脾化湿汤联合丁酸氢化可的松乳膏治疗特应性皮炎的疗效分析, 大家健康（下旬版） 9(10) (2016).

[57] 喻集保, 王琦, 李峧霓, 中西医结合治疗儿童特应性皮炎的疗效观察, 中国中医药科技 23(02) (2016) 219-220.

[58] 来亚群, 史小艳, 健脾渗湿法联合他克莫司治疗特应性皮炎疗效观察, 陕西中医 38(10) (2017) 1445-1446.

**Supplement 3**. Frequency of the components of oral HM in re-analyzed original randomized controlled trials

| **Latin name** | **Frequency** | **%** |
| --- | --- | --- |
| *Glycyrrhizae Radix et Rhizoma* | 39 | 70.91 |
| *Atractylodis Rhizoma Alba* | 37 | 67.27 |
| *Poria (Hoelen)* | 32 | 58.18 |
| *Angelicae Gigantis Radix* | 22 | 40.0 |
| *Dictamni Radicis Cortex* | 22 | 40.0 |
| *Atractylodis Rhizoma* | 19 | 34.55 |
| *Saposhnikoviae Radix* | 19 | 34.55 |
| *Citri Unshius Pericarpium* | 18 | 32.73 |
| *Astragali Radix* | 16 | 29.09 |
| *Coicis Semen* | 15 | 27.27 |
| *Tribuli Fructus* | 14 | 25.45 |
| *Rehmanniae Radix Crudus* | 14 | 25.45 |
| *Moutan Cortex Radicis* | 13 | 23.64 |
| *Alismatis Rhizoma* | 12 | 21.82 |
| *Dioscoreae Rhizoma* | 12 | 21.82 |
| *Schizonepetae Spica* | 11 | 20.0 |
| *Codonopsis Pilosulae Radix* | 8 | 14.55 |
| *Scutellariae Radix* | 8 | 14.55 |
| *Cicadae Periostracum* | 7 | 12.73 |
| *Anemarrhenae Rhizoma* | 7 | 12.73 |
| *Pseudostellariae Radix* | 7 | 12.73 |
| *Ostreae Testa* | 6 | 10.91 |
| *Paeoniae Radix Alba* | 6 | 10.91 |
| *Cnidii Rhizoma* | 6 | 10.91 |
| *Kochiae Fructus* | 6 | 10.91 |
| *Polygoni Multiflori Radix* | 6 | 10.91 |
| *Sophorae Radix* | 5 | 9.09 |
| *Lonicerae Flos* | 5 | 9.09 |
| *Lophatheri Herba* | 5 | 9.09 |
| *Persicae Semen* | 5 | 9.09 |
| *Liriopis Tuber* | 5 | 9.09 |
| *Spatholobi Caulis* | 4 | 7.27 |
| *Hordei Fructus Germinatus* | 4 | 7.27 |
| *Bupleuri Radix* | 4 | 7.27 |
| *Forsythiae Fructus* | 4 | 7.27 |
| *Arctii Semen* | 4 | 7.27 |
| *Paeoniae Radix Rubra* | 4 | 7.27 |
| *Uncariae Ramulus Et Uncus* | 4 | 7.27 |
| *Gardeniae Fructus* | 4 | 7.27 |
| *Salviae Miltiorrhizae Radix* | 4 | 7.27 |
| *Coptidis Rhizoma* | 4 | 7.27 |
| *Platycodonis Radix* | 3 | 5.45 |
| *Raphani Semen* | 3 | 5.45 |
| *Portulacae Herba* | 3 | 5.45 |
| *Sinomenii Caulis et Rhizoma* | 3 | 5.45 |
| *Dolichoris Semen* | 3 | 5.45 |
| *Adenophorae Radix* | 3 | 5.45 |
| *Crataegi Fructus* | 3 | 5.45 |
| *Gypsum Fibrosum* | 3 | 5.45 |
| *Rehmanniae Radix Preparata* | 3 | 5.45 |
| *Anisi Corii Colla* | 3 | 5.45 |
| *Polyporus* | 3 | 5.45 |
| *Trichosanthis Radix* | 3 | 5.45 |
| *Talcum* | 3 | 5.45 |
| *Phellodendri Cortex* | 3 | 5.45 |
| *Cinnamomi Ramulus* | 2 | 3.64 |
| *Jujubae Fructus* | 2 | 3.64 |
| *Junci Medulla* | 2 | 3.64 |
| *Akebiae Caulis* | 2 | 3.64 |
| *Pinelliae Tuber* | 2 | 3.64 |
| *Glehniae Radix* | 2 | 3.64 |
| *Cnidi Fructus* | 2 | 3.64 |
| *Cimicifugae Rhizoma* | 2 | 3.64 |
| *Zaocytis Caro Siccus* | 2 | 3.64 |
| *Polygonati Odorati Rhizoma* | 2 | 3.64 |
| *Fossilia Ossis Mastodi* | 2 | 3.64 |
| *Ginseng Radix* | 2 | 3.64 |
| *Scorpion* | 2 | 3.64 |
| *Rehmanniae Radix* | 2 | 3.64 |
| *Plantaginis Semen* | 2 | 3.64 |
| *Asparagus Tuber* | 2 | 3.64 |
| *Smilacis Rhizoma* | 2 | 3.64 |
| *Albizziae Cortex* | 2 | 3.64 |
| *Scrophulariae Radix* | 2 | 3.64 |
| *Carthami Flos* | 2 | 3.64 |
| *Tripterygium glycosides* | 2 | 3.64 |
| *Bombycis Corpus cum Batryticatus* | 1 | 1.82 |
| *Zingiberis Rhizoma* | 1 | 1.82 |
| *Galli Stomachichum Corium* | 1 | 1.82 |
| *Campsitis Flos* | 1 | 1.82 |
| *Arecae Pericarpium* | 1 | 1.82 |
| *Zizyphi Fructus* | 1 | 1.82 |
| *Araliae Continentalis Radix* | 1 | 1.82 |
| *Chaenomelis Fructus* | 1 | 1.82 |
| *Menthae Herba* | 1 | 1.82 |
| *Batryticatus Bombyx* | 1 | 1.82 |
| *Lilii Bulbus* | 1 | 1.82 |
| *Oldenlandiae Diffusae Herba* | 1 | 1.82 |
| *Arecae Semen* | 1 | 1.82 |
| *Dendrobii Herba* | 1 | 1.82 |
| *Polygoni Multiflori Caulis* | 1 | 1.82 |
| *Lini Semen* | 1 | 1.82 |
| *Polygoni Multiflori Ramulus* | 1 | 1.82 |
| *Nelumbinis Folium* | 1 | 1.82 |
| *Nelumbinis Semen* | 1 | 1.82 |
| *Scolopendrae Corpus* | 1 | 1.82 |
| *Schisandrae Fructus* | 1 | 1.82 |
| *Zaocys* | 1 | 1.82 |
| *Piperis Futokandsurae Caulis* | 1 | 1.82 |
| *Lithospermi Radix* | 1 | 1.82 |
| *Lycii Radicis Cortex* | 1 | 1.82 |
| *Xanthii Fructus* | 1 | 1.82 |
| *Meliae Fructus* | 1 | 1.82 |
| *Taraxaci Herba* | 1 | 1.82 |
| *Sesami Semen* | 1 | 1.82 |
| *Magnoliae Cortex* | 1 | 1.82 |
| *Angelicae Dahuricae Radix* | 1 | 1.82 |
| *Magnoliae Flos* | 1 | 1.82 |
| *Mori Radicis Cortex* | 1 | 1.82 |
| *Cynanchi Paniculati Radix* | 1 | 1.82 |
